# A Case of Plant Vaccination: Enhancement of Plant Immunity against *Verticillium dahliae* by Necrotized Spores of the Pathogen

**DOI:** 10.3390/plants11131691

**Published:** 2022-06-26

**Authors:** Eirini G. Poulaki, Maria Frantzeska Triviza, Marius Malai, Sotirios E. Tjamos

**Affiliations:** Laboratory of Plant Pathology, Agricultural University of Athens, 75 Iera Odos str., 11855 Athens, Greece; poulakie@gmail.com (E.G.P.); f.triviza@bpi.gr (M.F.T.); mariosmalai29@gmail.com (M.M.)

**Keywords:** CERK1, chitin, effector-triggered immunity, innate immune system, PAMP, PAMP-triggered immunity

## Abstract

The soil-borne fungus *Verticillium dahliae* is causing a devastating vascular disease in more than 200 species of dicotyledonous plants. The pathogen attacks susceptible plants through the roots, colonizes the plant vascular system, and causes the death of aerial tissues. In this study, we used *Arabidopsis* and eggplants to examine the plant protective and immunization effects of autoclaved *V. dahliae* spores against *V. dahliae*. We observed that the application of *V. dahliae* autoclaved spores in eggplants and *Arabidopsis* resulted in enhanced protection against *V. dahliae*, since the disease severity and pathogen colonization were lower in the plants treated with *V. dahliae* autoclaved spores when compared to controls. In addition, upregulation of the defense related genes *PR1* and *PDF1.2* in the *Arabidopsis* plants treated with the *V. dahliae* autoclaved spores was revealed. Furthermore, pathogenicity experiments in the *Arabidopsis* mutant *cerk1*, defective in chitin perception, revealed a loss of protection against *V. dahliae* in the *cerk1* treated with the *V. dahliae* autoclaved spores. The participation of the chitin receptor CERK1 is evident in Arabidopsis immunization against *V. dahliae* using autoclaved spores of the pathogen.

## 1. Introduction

Plants possess an innate immune system comprised of pathogen associated molecular pattern (PAMP)-triggered immunity (PTI) and effector-triggered immunity (ETI). PAMPs are conserved molecular patterns in pathogens, alarming plants of the presence of potential intruders in their close vicinity [1]. Chitin, as one of the most conserved domains in the fungal cell wall, is a well-studied PAMP that could be sensed by plants, which in turn activate their defense mechanisms [2]. Chitin is a polysaccharide [β-1,4-N-acetylglucosamine (GlcNAc)n], which can be degraded to smaller parts, oligosaccharides, due to the action of plant chitinases [3]. Subsequently, the generated chitin fragments can be recognized by a chitin perception system resulting in the activation of PTI [2]. The plant chitin-sensing system comprises of receptors belonging to the lysin motif (LysM) domain family, including pattern recognition receptors (PRRs), receptor-like kinases (RLKs), and receptor-like proteins (RLPs) [4]. In *Arabidopsis thaliana*, the LysM-RLK chitin receptor CERK1 has been identified to bind N-acetylated chitin fragments, triggering the downstream plant defense [5,6].

An intriguing aspect of plant defense is the phenomenon of priming. This is a plant immunological state triggered by pathogen derived compounds, effectors, beneficial microbes, and various synthetic/natural compounds which enable plants to display a timely and robust activation of plant defenses upon detection of a biotic or abiotic stress [7,8,9,10]. In these respects, various biocontrol agents have been used to trigger induced systemic resistance in plants against various plant pathogens, mainly against plant pathogens where no effective chemical controls exist, such as *Verticillium dahliae* [11].

The soilborne fungal pathogen *V. dahliae* infects over 200 dicotyledon plant species, causing significant losses in plant yield, mostly in temperate zones [12,13]. The pathogen infects plants through the root system and spreads through the plant causing leaf wilting, chlorosis, yellowing, and tissue discoloration, eventually leading to plant death. The endophytic lifestyle of the pathogen and the production of long-living dormant structures, microsclerotia, make the pathogen inaccessible to chemical compounds. In addition, the scarcity of resistance genes in plant species poses a further limitation to *Verticillium* disease control [14]. Therefore, most research has focused on the development of biocontrol-based disease management strategies. However, these strategies are time consuming and impose a microbial input in the soil environment which may have detrimental effects to the indigenous microbiome and could be ineffective since the introduced microorganisms have to overcome various biotic and abiotic stresses under field conditions. 

With these considerations, we investigated the potential of autoclaved *V. dahliae* spores to prime *Arabidopsis thaliana* and eggplants against *V. dahliae*. The priming effect of necrotized pathogen spores has not attracted any attention until now and to our best knowledge, this is the first published study describing the efficacy of necrotized pathogen structures to trigger the plant defense mechanism against a pathogen. The autoclaved *V. dahliae* spores were applied to the plants either as a root drenching or stem/leaf injecting inoculum. The observed plant protective activity of the *V. dahliae* spores led us to further investigate (i) the expression levels of the plant defense genes, *PR1* and *PDF1.2*, marker genes of the salicylic acid (SA) and ethylene/jasmonate (ET/JA) dependent defenses [15], respectively and (ii) the participation of the CERK1 receptor using the *A. thaliana* mutant *cerk1-2* [16]. 

## 2. Results

### 2.1. The Autoclaved Spores of V. dahliae Protect A. thaliana and Eggplants against V. dahliae

**Figure 1:**
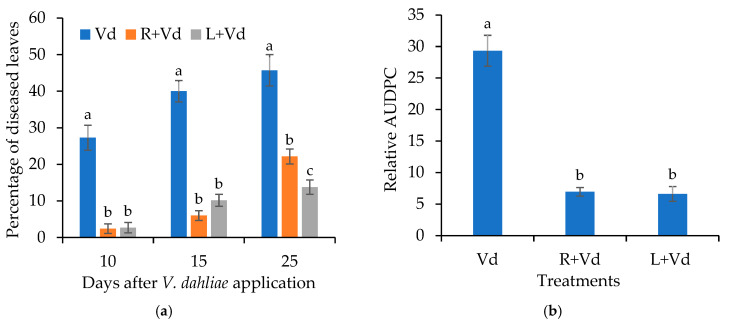
Disease severity (**a**) and relative AUDPC (**b**) caused by artificial inoculation of *V. dahliae* (Vd), on *Arabidopsis thaliana* leaf injected (L + Vd) or root drenched (R + Vd) with autoclaved spores of *V. dahliae* or mock inoculated (positive control, Vd). Columns represent means of three replications per treatment (*n* = 30 plants, ± SE). Different letters denote statistically significant differences between treatments (*p* < 0.05) according to LSD test. For disease severity, statistics were performed at each time point.

The addition of autoclaved spores of *V. dahliae* reduced *Verticillium* wilt symptoms in *A. thaliana* Col-0. The Verticillium symptoms appeared mainly in the form of wilting. The disease severity of *Verticillium* was recorded from 10 days post-inoculation (dpi) to 25 dpi (Figure 1). 

The *A. thaliana* plants treated with the autoclaved spores of *V. dahliae* showed a significantly lower amount of disease during the pathogenicity experiment. At the last recording (25 dpi), the percentage of diseased leaves was ca. 14% and 22% in the leaf-injected and root-treated plants with the autoclaved spores of *V. dahliae*, respectively, while in the controls, the percentage of diseased leaves was 46%. The analysis of the relative area under the disease progress curve (AUDPC) values verified that the overall amount of the disease severity in the plants treated with the autoclaved spores of the pathogen was lower than the control.

**Figure 2:**
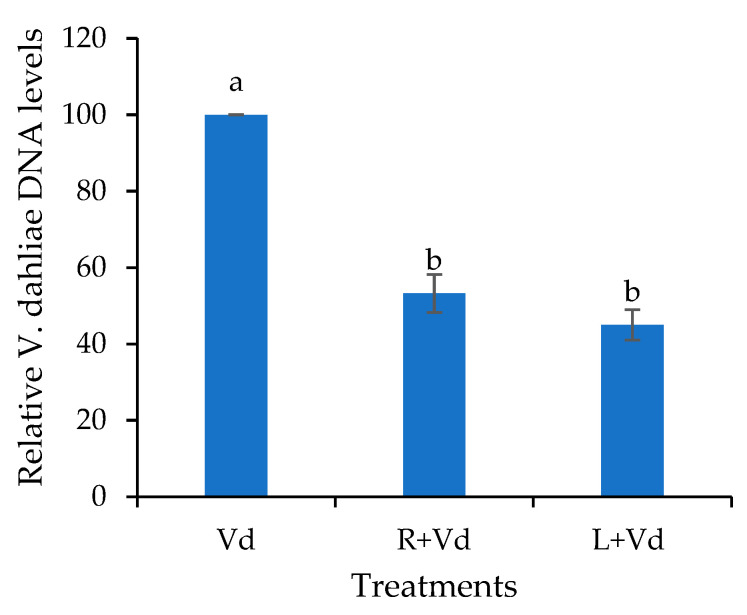
Relative quantification of the *V. dahliae* DNA levels in wild-type *A. thaliana* leaf injected (L + Vd) or root drenched (R + Vd) with autoclaved spores of *V. dahliae* or mock inoculated (positive control, Vd) and inoculated after 3 days with *V. dahliae*. The relative *V. dahliae* DNA levels were estimated by qPCR using total DNA isolated from the aerial parts of plants at 25 days post-inoculation. Columns represent means of three biological and technical replications per treatment (*n* = 9, ± SE) at 25 dpi (end of pathogenicity experiment). Different letters denote statistically significant differences between treatments (*p* < 0.05) according to LSD test.

The disease severity results were confirmed by qPCR analysis of the endophytic DNA levels of the pathogen in the different treatments. It was shown that the *A. thaliana* plants that have been treated with the autoclaved spores of *V. dahliae* harbored significantly lower relative *V. dahliae* DNA levels than the controls (Figure 2). Additionally, a statistically significant difference between the plant protective treatments was not recorded. Therefore, treating plants with necrotized propagules of the pathogen leads to plant protection.

**Figure 3:**
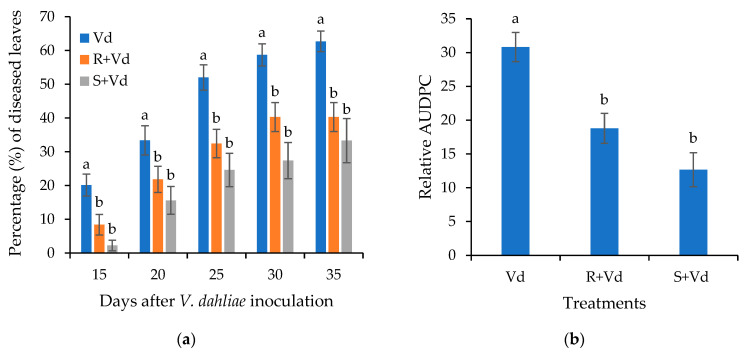
*Verticillium* disease severity (**a**) and relative AUDPC (**b**) caused by artificial inoculation of *V. dahliae* (Vd), on eggplants stem injected (S + Vd) or root drenched (R + Vd) with autoclaved spores of *V. dahliae* or mock inoculated (positive control, Vd). Columns represent means of three replications per treatment (*n* = 30 plants, ± SE). Different letters denote statistically significant differences between treatments (*p* < 0.05) according to LSD test. For disease severity, statistics were performed at each time point.

The plant protective activity of the autoclaved spores of *V. dahliae* was also examined in eggplants. As in *A. thaliana*, the initial disease symptoms appeared in the form of wilting (Supplementary Figure S1). Symptom recording started at 15 dpi and lasted until 35 dpi (Figure 3). The eggplants treated with autoclaved spores of *V. dahliae* had less *Verticillium* wilt symptoms than the controls during the experiment. At the last recording (35 dpi), the percentage of diseased leaves was ca. 33% and 40% in the leaf-injected and root-treated plants with the autoclaved spores of *V. dahliae*, respectively, while in the controls the percentage of diseased leaves was 63%. The analysis of the relative AUDPC values verified that the overall level of disease severity in the plants treated with the autoclaved spores of the pathogen was lower than the control.

### 2.2. The Autoclaved Spores of V. dahliae Trigger the Plant Defence Mechanisms against V. dahliae

The plant pathogenicity experiments revealed that treating *A. thaliana* and eggplants with autoclaved spores of *V. dahliae* leads to a significant reduction in disease severity when compared to the control. Therefore, we wanted to investigate whether certain defense-related genes were upregulated in the plant protective treatments. For this purpose, we investigated the expression levels of the plant defense genes *PR1* and *PDF1.2*, marker genes of the SA and ET/JA dependent defenses, at 3 and 7 dpi. 

**Figure 4:**
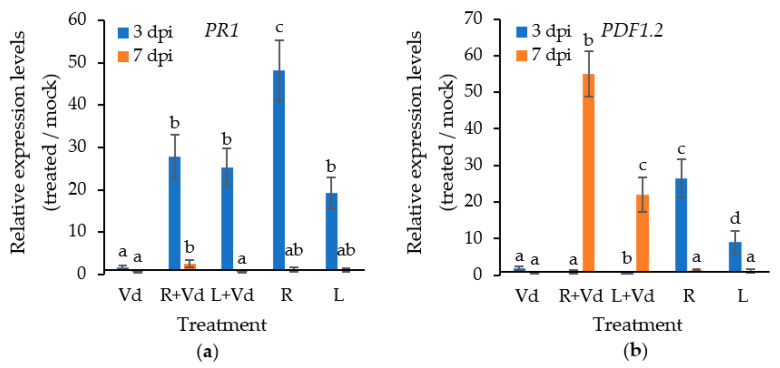
Relative transcript levels of *PR1* (**a**) and *PDF1.2* (**b**) in the aerial plant parts of *A. thaliana* leaf injected (L + Vd) or root drenched (R + Vd) with autoclaved spores of *V. dahliae* or mock inoculated (positive control, Vd) in response to infection with *V. dahliae* at 3 and 7 dpi. The samples for RNA isolation were collected at 3 and 7 days post inoculation (dpi) with the pathogen. The expression levels of *PR1* and *PDF1.2* were normalized to the expression of the reference gene *At4g26410* measured in the same samples and they are presented in comparison to the normalized expression level of the respective gene in the mock treatment. Columns represent means of three biological and technical replications per treatment (*n* = 9, ± SE). At each day, different letters denote statistically significant differences between treatments (*p* < 0.05) according to LSD test.

The application of autoclaved spores of *V. dahliae* upregulated the expression of *PR1* and *PDF1.2* at 3 dpi; however, this was a transient phenomenon, since the expression of both genes became similar to the mock-inoculated samples at 7 dpi (Figure 4). Upon pathogen inoculation, the expression of *PR1* and *PDF1.2* followed opposite patterns, since the expression of *PR1* was higher at 3 dpi than 7 dpi, while the expression of *PDF1.2* was higher at 7 dpi than at 3 dpi in the plants treated with the autoclaved spores of *V. dahliae*, irrespective of the application method. The expression of *PR1* was upregulated by 22 folds in the plants treated with the autoclaved *V. dahliae* spores compared to mocks at 3 dpi, while *PDF1.2* was upregulated by 22- and 55-fold in the leaf-injected and root-drenched plants with the autoclaved spores of *V. dahlia* at 7 dpi, respectively. In the control treatment (Vd), the expression of *PR1* and *PDF1.2* was upregulated at 3 dpi by 1.7- and 2-fold when compared to the mock-inoculated plants, respectively, and downregulated at 7 dpi. 

### 2.3. The Plant Protective Activity of the Autoclaved V. dahliae Spores Depends on CERK1

Having observed the plant protective activity of the autoclaved spores of *V. dahliae* and the upregulation of defense-related genes in these treatments upon *V. dahliae* infection, we investigated whether the chitin receptor CERK1 is involved in the suggested PAMP-triggered immunity against *V. dahliae*. For this purpose, we investigated the CERK1 levels in wild-type *A. thaliana* treated with autoclaved spores of *V. dahliae* upon *V. dahliae* infection at 3 and 7 dpi and the plant protective activity of the autoclaved spores of *V. dahliae* in the *A. thaliana* mutants *cerk1*.

**Figure 5:**
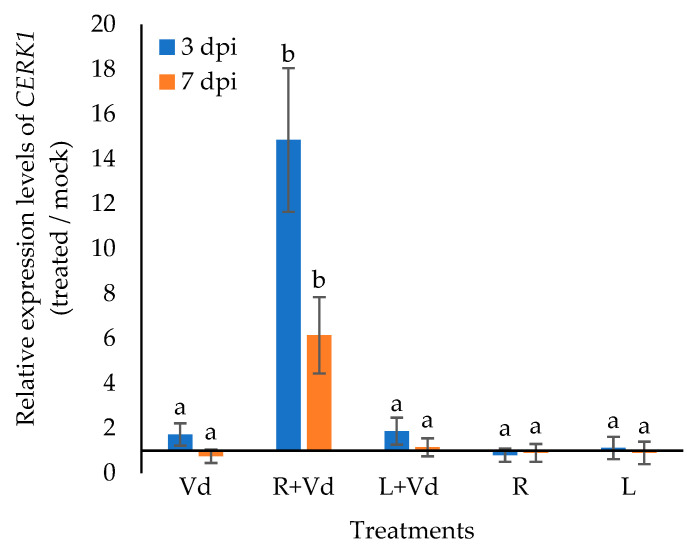
Relative transcript levels of *CERK1* in the aerial plant parts of *A. thaliana* leaf injected (L + Vd) or root drenched (R + Vd) with autoclaved spores of *V. dahliae* or mock inoculated (positive control, Vd) in response to infection with *V. dahliae*. The samples for RNA isolation were collected at 3 and 7 days post inoculation (dpi) with the pathogen. The expression levels of *CERK1* were normalized to the expression of the reference gene *At4g26410* measured in the same samples and they are presented in comparison with the normalized expression level of the respective gene in the mock treatment. Columns represent means of three biological and technical replications per treatment (*n* = 9, ± SE). At each day, different letters denote statistically significant differences between treatments (*p* < 0.05) according to LSD test.

Indeed, in the case of the root-drenched autoclaved spores of *V. dahliae*, the expression of *CERK1* was upregulated by 15- and 6-fold when compared to the mock treatment at 3 and 7 dpi, respectively (Figure 5). The application of the autoclaved spores of *V. dahliae* in the leaves resulted in the marginal upregulation of *CERK1* upon *V. dahliae* infection, similar to the control treatment (*V. dahliae*) at 3 dpi. On the contrary, the expression levels of *CERK1* were not upregulated in the absence of infection. The importance of *CERK1* in the plant protective activity of the autoclaved spores of *V. dahliae* was further examined in *cerk1*.

**Figure 6:**
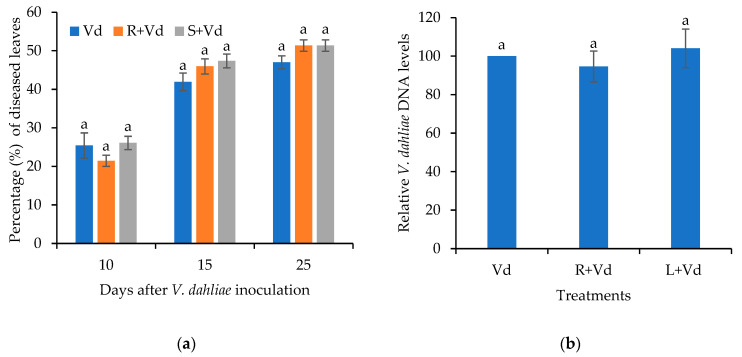
Disease severity and relative quantification of the *V. dahliae* DNA levels in *A. thaliana*, *cerk1*, leaf injected (L + Vd) or root drenched (R + Vd) with autoclaved spores of *V. dahliae* or mock inoculated (positive control, Vd) and inoculated after 3 days with *V. dahliae*. Fungal DNA levels were estimated at 25 dpi. In (**a**), columns represent means of three replications per treatment (*n* = 30 plants, ± SE) and in (**b**) they represent means of three biological and technical replications per treatment (*n* = 9, ± SE) at 25 dpi (end of pathogenicity experiment). In (**b**), the control treatment (Vd) is set to 100%. Different letters denote statistically significant differences between treatments (*p* < 0.05) according to LSD test. For disease severity, statistics were performed at each time point.

Such as in the previous pathogenicity experiments, the initial *Verticillium* symptoms in *cerk1* were wilting, followed by leaf yellowing and necrosis. Symptom recording started at 10 days post inoculation (dpi) and lasted until 25 dpi (Figure 6). In contrast to the wild-type *A. thaliana*, the application of the autoclaved spores of *V. dahliae* in the *cerk1* plants did not result in reduced diseased severity in comparison to the control. In agreement with the pathogenicity experiments, the qPCR analysis revealed that the endophytic *V. dahliae* DNA levels were similar across the different treatments (Figure 6). 

## 3. Discussion

*Verticillium* wilt, mainly caused by *V. dahliae*, is a devastating disease throughout the world, causing significant losses in plant yield every year. The lack of chemical control and the scarcity of plant resistance genes has boosted research efforts to identify novel disease management strategies [14]. Our results showed that the application of autoclaved spores of *V. dahliae* in *A. thaliana* and eggplants resulted in a significant reduction of *Verticillium* symptoms when compared to controls. According to our best knowledge, this is the first report showing the plant protective activity of necrotized spores of a pathogen against its active live form. This disease control approach resembles the vaccination effects in mammals, where inactive forms of a pathogen are used to promote immunity. 

As a concept, plant vaccination is gaining increasing grounds over the last decades, mainly using natural or synthetic molecules that stimulate plant immunity [17]. These molecules are recognized by plants as either damage-associated molecular patterns (DAMPs) or PAMPs [18]. In our case, the molecules of the autoclaved *V. dahliae* spores fell into the second category, PAMPs, with the most prominent and well-studied molecule to be chitin. Our results suggest that eggplants and *A. thaliana* perceived the *V. dahliae* autoclaved spores as PAMPs and elicited their immune responses as a priming effect. Primed plants display timely and robust triggering of their defense responses upon biotic or abiotic stress [7,8,9,10]. Indeed, the treatment of *A. thaliana* plants with the autoclaved *V. dahliae* spores resulted in the upregulation of the examined defense genes *PR1* and *PDF1.2* upon pathogen infection.

The plant defense mechanism comprises of two main branches, depending on SA and ET/JA. The SA dependent defense mechanism is active against biotrophic pathogens, while the ET/JA defense branch is triggered against necrotrophs [19]. *V. dahliae* is considered as a hemibiotroph, having a biotrophic life style at the early infection stages, so as to establish itself in the xylem vessels, then switches to necrotrophism, producing various compounds toxic to the plant, leading to plant death [20,21]. This dual life style was anticipated in the expression of the defense genes *PR1* and *PDF1.2* in the pathogen inoculated *A. thaliana* that have been treated with the *V. dahliae* autoclaved spores, since *PR1* expression was higher at 3 dpi than 7 dpi while the expression of *PDF1.2* was higher at 7 dpi than 3 dpi. The opposite expression patterns between *PR1* and *PDF1.2* also anticipates the existing antagonism between the SA and the ET/JA defense mechanisms [19]. This antagonism protects plants from a fitness cost caused by the allocation of metabolic resources toward defense that would otherwise have been allocated to growth and reproduction [19]. Therefore, the early upregulation of *PR1* may be crucial for plant defense against the biotrophic phase of the pathogen, while the later upregulation of *PDF1.2* may target the necrotrophic phase. In the past, Thaler et al. (2004) [20] suggested a critical role of the JA-dependent defenses against *V. dahliae*, since the jasmonate-deficient tomato mutant plants, *def1*, were more susceptible than the wild type. Furthermore, exogenous application of SA and ectopic activation of the JA pathway provided am increased resistance to *A. thaliana* against the hemibiotrophic vascular pathogen *Fusarium oxysporum*, which has a similar disease pattern to *V. dahliae* [22]. 

A main component of the fungal cell wall is chitin. The plants, having evolutionarily anticipated the presence of chitin in their potential fungal enemies, were equipped with the plasma membrane *CERK1* receptor, which binds chitin in association with LYK5 in *A. thaliana* [4,5,6]. In our experiments, the plant protective activity of the *V. dahliae* autoclaved spores was lost in the *cerk1* mutants, suggesting the importance of the *CERK1* receptor in the interaction of plants with the *V. dahliae* autoclaved spores and the concomitant triggering of plant defenses. In other studies, the treatment of plants or their cell cultures with chitin oligomers elicited various defense responses, such as synthesis of PRs and phytoalexins [23,24]. Interestingly, after being identified as a chitin receptor, *CERK1* was also identified as a receptor for linear 1,3-b-D-glucans, a major component of the fungal and oomycete cell wall. *CERK1* was also identified as having a role in the perception of bacterial peptidoglycans in Arabidopsis [25]. Since chitin represents a smaller proportion of the fungal cell walls than glucans [25], glucans may have played a stronger role than chitin in the observed plant protection against *V. dahliae*. Overall, the potential PAMPs sensed by eggplants and *A. thaliana* are chitin and glucans. An interesting decoupling was observed between the two application methods of the autoclaved *V. dahliae* spores, since the expression of *CERK1* was upregulated in the plants that have been root drenched by the *V. dahliae* autoclaved spores upon pathogen infection, while the leaf infiltration application did not result in the upregulation of *CERK1*. A possible explanation is the occurrence of higher ligand availability in the case of root drenching when compared to leaf infiltration, since 10 milliliters of *V. dahliae* autoclaved spores were applied per plant in the root-drenching application, compared to 20 microliters in the case of leaf infiltration. Nevertheless, the plant protection levels were statistically identical between the two treatments. Therefore, the expression levels of *CERK1* did not influence the observed plant protection, suggesting the need for a functional *CERK1* rather than a multicopy *CERK1* in the triggering of immune responses against *V. dahliae*.

In conclusion, our results show the potential of necrotized spores of a pathogen to protect plants against its active form. It remains an open question for further studies whether the use of pure chitin, glucan, or other PAMPs could offer a higher level of protection against *V. dahliae* when compared to the autoclaved spores. However, we can suggest that the use of the *V. dahliae* propagules may offer an array of PAMPs that trigger and more effectively shape the defense mechanisms in a larger group of plant species than a single PAMP. Since it is known that some PAMPs are perceived by a limited number of plant species while others activate defense responses in a broad range of species [26], the use of autoclaved spores may offer protection against a large group of plant species. Overall, this study constitutes the first described attempt in the scientific literature to control *V. dahliae* through plant vaccination using its own propagating structures. The results suggest the involvement of the *CERK1* receptor and the concomitant triggering of the plant defense genes *PR1* and *PDF1.2* in a manner that resembles the biotrophic and necrotrophic disease pattern of *V. dahliae*.

## 4. Materials and Methods

### 4.1. Verticillium dahliae Culture

A *V. dahliae* isolate, originated either from infected *Raphanus sativus* L. or *Solanum melongena* L. were used in the *A. thaliana* and eggplant bioassays, respectively [27,28]. A suspension of 10^7^ *V. dahliae* conidia/mL of distilled sterile water was prepared from a culture grown for 5 days at 24 °C in a sucrose sodium nitrate liquid medium [29]. 

### 4.2. Seeds Origin and Plant Growth Conditions

Wild-type *A. thaliana* Col-0 and the *cerk1-2* mutant were provided by J. P. Métraux (University of Fribourg, Switzerland). For the experiments, *A. thaliana* and eggplant were grown separately in pots (9 × 9 × 10 cm) containing pasteurized soil. The plant growth conditions were: 25 °C, 16 h photoperiod, and 60–70% relative humidity.

### 4.3. Pathogenicity Experiments

Twenty-day-old wild-type *A. thaliana,* Col-0 plants were either root drenched with 10 mL or leaf injected with 10 μL of 10^7^ autoclaved (121 °C, 1.2 atm, 15 min) *V. dahliae* conidia mL^−1^ sterile distilled water per plant or sterile distilled water (mock treatments). After 3 days, the plants were root drenched with 10 mL of 10^7^ *V. dahliae* conidia mL^−1^ sterile distilled water per plant or sterile distilled water (mocks, negative control) [11]. The experiment was repeated three times with 10 plants per treatment per replication (a total of 30 plants per treatment). Disease severity was calculated as a percentage of the leaves showing *Verticillium* symptoms over the total number of leaves of each plant. Subsequently, the relative AUDPC value, the percentage of the AUDPC value over the maximum possible AUDPDC for the experimental time period, was calculated for each plant [30]. The same methodology was followed for the pathogenicity experiments using the *A. thaliana,* Col-0, *cerk1-2* mutants.

The evaluation of the plant protective activity of *V. dahliae* necrotized spores against *V. dahliae* on eggplants was performed by root drenching 10 mL or stem injecting 10 μL of 10^7^ autoclaved (121 °C, 1.2 atm, 15 min) *V. dahliae* spores mL^−1^ sterile distilled water per plant and following the same methodology as it is described above.

### 4.4. DNA extraction and qPCR V. dahliae quantification

In the *A. thaliana* bioassays (wt and *cerk1-2*), the relative *V. dahliae* DNA endophytic levels of the different treatments were quantified at 25 dpi. Total DNA was isolated from the aerial plant parts of 10 plants per treatment, pooled to one sample and ground to fine powder using liquid nitrogen. The extraction of DNA was performed according to the methodology of Dellaporta et al. (1983) [31]. qPCRs were performed in an Applied Biosystems StepOnePlus thermocycler and for the amplification reactions, KAPA SYBR^®^ FAST (Merck SA) was used. The primers used for real-time PCR were ITS1-F 5–AAAGTTTTAATGGTTCGCTAAGA–3 and ST-VE1-R 5–CTTGGTCATTTAGAGGAAGTAA–3 for the ITS1 and ITS2 regions of the 5.8S ribosomal RNA gene (Z29511) of *V. dahliae*. [11] and 5-GAGCTGAAGTGGCTTCCATGAC-3 and 5-GGTCCGACATACCCATGATCC-3 for the reference *Arabidopsis* gene *At4g26410* [32]. PCR efficiency for each amplicon was calculated by employing the linear regression method on log (fluorescence) per cycle number data, using the Lin-Reg PCR software [33]. For each treatment, three biological repeats and three technical repeats per biological repeat were conducted. The absence of nonspecific products and primer dimers was confirmed by analysis of the melting curves. The relative *V. dahliae* DNA quantity, calculated by using the formula 2^−∆Ct^, was expressed as a percentage compared to the wild type; while the value (2^−∆Ct^) of the wild type was set to 100% [11].

### 4.5. Determination of PR1, PDF1.2 and CERK1 transcript levels

For RNA analysis, the aerial parts of five *A. thaliana* wt plants from each treatment and experimental replication (a total of 3 replications) were collected and pooled to one sample at 3 and 7 dpi. The pooled tissues were ground with liquid nitrogen and total RNA was extracted by using TRIzol^®^ Reagent (Invitrogen, Paisley, Renfrewshire, UK), according to the manufacturer’s instructions. The RNA samples were treated with DNase I (Macherey-Nagel) to eliminate traces of contaminating genomic DNA. First-strand cDNA was synthesized using PrimeScript One Step RT-PCR Kit (Takara) following the manufacturer’s procedure. qPCRs were performed in an Applied Biosystems StepOnePlus thermocycler and for the amplification reactions, KAPA SYBR^®^ FAST (Merck SA) was used. The primers used for real-time PCR were *CERK1* (AT3G21630; 5-CAAATCAAGAGATGGTGTTGGTGC-3 and 5- CACCACCCAAACCTCCACT-3) [34], *PR1* (AT2G14610; 5-GTGCCAAAGTGAGGTGTAACAA-3 and 5-CGTGTGTATGCATGATCACATC-3) and *PDF1.2* (AT5G44420; 5-TTTGCTGCTTTCGACGCAC-3 and 5-CGCAAACCCCTGACCATG-3) [35]. PCR efficiency for each amplicon was calculated by employing the linear regression method on log (fluorescence) per cycle number data, using the Lin-Reg PCR software [33]. For each treatment, three biological repeats and three technical repeats per biological repeat were conducted per sampling time point (3 and 7 dpi). The absence of nonspecific products and primer dimers was confirmed by analysis of the melting curves. The relative *PR1, PDF1.2,* and *CERK1* expression levels (2^−∆Ct^) in the treated plants were expressed relative to the normalized transcript levels of *PR1*, *PDF1.2* and *CERK1* in the mock-inoculated plants (negative control).

### 4.6. Statistics

Data on disease severity, relative AUDPC, relative *V. dahliae* DNA levels, and gene expression were subjected to analyses of variance. When a significant (*p* < 0.05) F-test was obtained for the treatments, data were analyzed by LSD multiple range test (*p* < 0.05). 

## Data Availability

The data presented in this study are available on request from the corresponding author.

## Supplementary Materials

The following supporting information can be downloaded at: https://www.mdpi.com/article/10.3390/plants11131691/s1, Figure S1: *Verticillium* wilt symptoms caused on stem-injected (a) or root-drenched (b) eggplants with autoclaved spores of *V. dahliae*, controls (*V. dahliae* inoculated) (c) and mock-inoculated plants (d). The photograph was taken at 25 dpi.
