# Supplementary material for: A Case of Plant Vaccination: Enhancement of Plant Immunity against Verticillium dahliae by Necrotized Spores of the Pathogen
Source: Plants (Basel). 2022 Jun 26;11(13):1691. doi: 10.3390/plants11131691 (PMC9269021; doi:10.3390/plants11131691)
Supplement: Supplementary file 1 [file plants-11-01691-s001.zip › plants-1767836-supplementary.pdf]

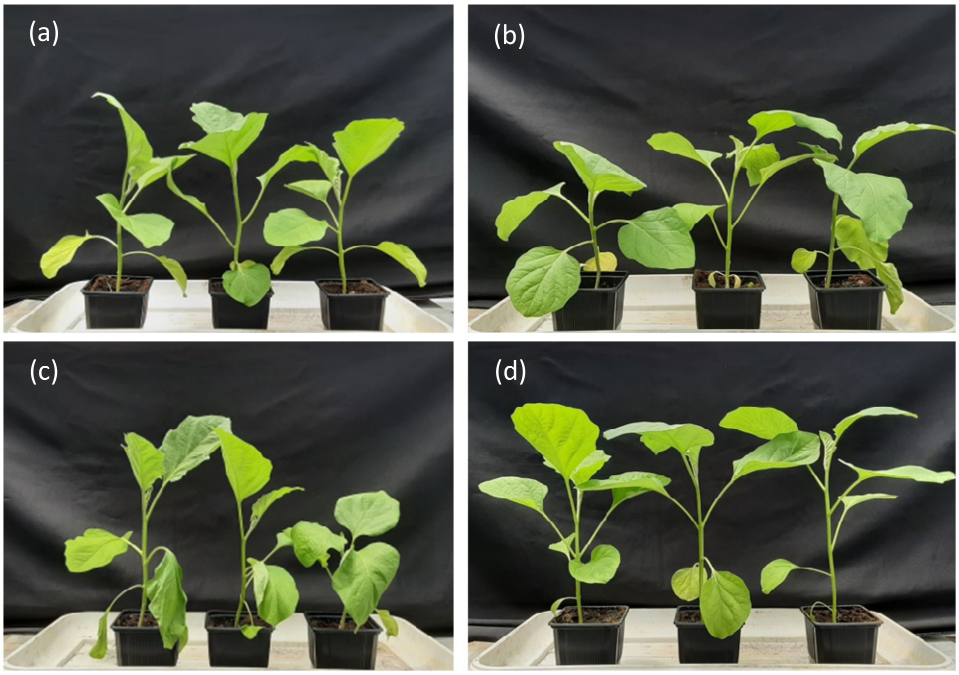

Supplementary Figure S1: Verticillium wilt symptoms caused on eggplants stem injected (a) or root drenched (b) with autoclaved spores of *V. dahliae*, controls (*V. dahliae* inoculated) (c) and mock inoculated plants (d). The photograph was taken at 25 dpi.
